# Urolithiasis, Independent of Uric Acid, Increased Risk of Coronary Artery and Carotid Atherosclerosis: A Meta-Analysis of Observational Studies

**DOI:** 10.1155/2020/1026240

**Published:** 2020-02-20

**Authors:** Wei Luo, Yao Zhou, Chenlin Gao, Pijun Yan, Ling Xu

**Affiliations:** ^1^Southwest Medical University, Luzhou 646000, China; ^2^Department of Endocrinology, Affiliated Hospital of Southwest Medical University, Luzhou 646000, China

## Abstract

**Background and Aims:**

Recent epidemiological evidence indicates an association between urolithiasis and atherosclerosis; however, results are incongruous. Our aim is to summarize the association between urolithiasis and arteriosclerosis risk through a detailed meta-analysis.

**Methods:**

Relevant studies published before April 2019 were identified by searching OVID, EMBASE, PubMed, Web of Science database, and Cochrane Library. The relationship between urolithiasis and the risk of atherosclerosis was assessed by using odds ratio (OR) values and the corresponding 95% confidence intervals (CIs), and the selection of fixed- or random-effects model based on heterogeneity.

**Results:**

The meta-analysis includes 8 observational studies that contained 70,716 samples. Pooled results showed that urolithiasis was associated with an increased adjusted and unadjusted risk estimated for atherosclerosis (*P*=0.017 and *P*=0.017 and *n* = 65,751/70,716) with serum uric acid levels less than 6.0 mg/dl, it still showed that urolithiasis was associated with a higher risk of atherosclerosis (*P*=0.017 and *I*^2^ = 0.0%, *P*=0.017 and *P*=0.017 and *P*=0.017 and

**Conclusions:**

Urolithiasis is associated with an increased risk for atherosclerosis, especially in coronary artery and carotid atherosclerosis. Urolithiasis may be another potential risk factor of atherosclerosis, which is independent of serum uric acid levels.

## 1. Introduction

Urolithiasis, especially kidney stones, is a common urinary tract disease. Seklehner et al. pointed out that the absolute number of patients treated for (+31.5%) and diagnosed with (+85.1%) renal calculus increased from 2001 to 2010 [1]. Researches in recent years have shown that the incidence of urolithiasis has increased not only in Asian countries [2] but also in European [3–5] and American countries [6]. An association between urolithiasis and metabolic syndrome [7–9] has been shown in many epidemiological studies, including hypertension, diabetes mellitus [8], hyperuricemia, obesity [10], hypercholesterolemia, and chronic kidney disease. Some studies have shown that urolithiasis is closely related to an increased risk of cardiovascular diseases [11], which include coronary heart disease [12], myocardial infarction [11], and stoke [13]. As is known to all, metabolic disorders are also risk factors for atherosclerosis [14–16]. It has also been indicated that urolithiasis and atherosclerosis share the common underlying risk factors [17–19]. Epidemiological studies have reached different results on the association between urolithiasis and the risk of arteriosclerosis in the confidence interval. Some studies have demonstrated a positive relationship [20–24], while others have not reported a significant correlation [25–27]. Since urolithiasis is a common urinary tract disease and atherosclerosis can bring patients grave consequences, our aim is to sum up the relationship between urolithiasis and arteriosclerosis risk in the observational studies through the meta-analysis. Further understanding of this association can draw attention of clinicians to provide early intervention.

### 1.1. Data Sources and Search Strategy

We systematically searched EMBASE, PubMed, OVID, Web of Science database, Cochrane Library, and conference proceedings (last update in April 2019). The following Medical Subject Headings (MeSH) search terms and keywords were used: “urolithiasis” or “urinary tract stone” or “renal stones” or “bladder calculi, urinary” or “vesical calculi” or “calculi, vesical” or “bladder calculi” or “cystoliths” or “calculi, ureteral” and “atherosclerosis” or “arterioscleroses” or “artery diseases” or “coronary atherosclerosis” or “aortic atherosclerosis” or “abdominal aorta atherosclerosis” or “carotid atherosclerosis” or “iliac artery atherosclerosis” or “cardiovascular disease” or “cardiovascular events.” No language restrictions were imposed.

### 1.2. Study Selection and Selection Criteria

Studies which are in compliance with the following inclusion criteria were enrolled: (1) the study with an observational study design, (2) the study provided with the OR (odds ratios) value with the corresponding 95% CIs (confidence intervals) for events associated with urolithiasis versus without urolithiasis, and (3) the study that assessed the incidence of atherosclerotic vascular abnormalities (defined as abdominal aortic calcification, carotid atherosclerosis, coronary artery calcification (CAC), peripheral aortic calcification, and increased arterial stiffness) among patients with urolithiasis. Animal experiment, case reports, editorials, literature reviews, and comments were excluded. Two reviewers (WL and PY) confirmed the adequacy of studies based on predefined inclusion criteria for titles and abstracts. For each eligible study included, the 2 reviewers assessed the full text. Discrepancies in data extraction were resolved through consensus.

### 1.3. Data Extraction

Two reviewers (WL and YZ) extracted data and assessed the accuracy. The differences were resolved by consensus and arbitration of the correspondent author (LX). The following information was obtained: author's name, year of publication, demography of the subjects (sex, samples, age, and country), follow-up duration, number of arteriosclerosis events in people with and without urolithiasis, analysis of arteriosclerosis events, adjustment confounders, outcome, outcome measurement, adjusted and unadjusted OR values and their 95% CIs, results, and the basic characteristics of the study sample (median body mass index (BMI), glucose, serum creatinine, uric acid, triglyceride (TC), low-density lipoprotein cholesterol (LDL-C), estimated glomerular filtration rate (eGFR), high-density lipoprotein cholesterol (HDL-C), and systolic and diastolic blood pressures) of each study.

### 1.4. Quality Assessment

The methodological quality of 4 cohort studies, 2 case-control studies, and 2 cross-sectional studies was assessed by 2 authors independently (YZ and LX) using the Newcastle–Ottawa scale and AHRQ scale. The cohort studies and case-control studies were scored according to the three major categories of the NOS (selection, comparability, and assessment of the outcome). The maximum scores for selection, comparability, and exposure were 4, 2, and 3, respectively. The quality of overall case-control studies and cohort studies was arbitrarily defined as fair to good (score, 4–8) or poor (score, <3). The methodological quality of the cross-sectional studies included was assessed by an 11-item checklist which was recommended by the Agency for Healthcare Research and Quality (AHRQ). The quality of overall cross-sectional studies was arbitrarily defined as fair to good (score, 4–11) or poor (score, <3).

### 1.5. Data Synthesis and Statistical Analysis

The OR value and its 95% CI were calculated to assess the association between urolithiasis and the incidence of atherosclerosis. Log OR values were combined in the statistical analysis.

The effect model was selected according to the size of heterogeneity. If heterogeneity was greater than 50%, the random-effects model of the inverse-variance method would be used to pool OR values with their 95% CI; otherwise, the fixed-effects model of the inverse-variance method would be chosen. To detect heterogeneity, we used Cochran's Q test, and *I*^2^ statistic (*I*^2^ > 75% indicated high heterogeneity) was used for quantification. To test whether the results of the study would be different or not, BMI subgroup and outcome subgroup analyses were carried out, and the causes of heterogeneity could be further analyzed. The data analysis was performed by STATA, version 11.0. The double-tail *P* value less than 0.05 was statistically significant.

### 1.6. Publication Bias and Sensitivity Analysis

To evaluate the influence of each study on the overall effect size, we conducted a sensitivity analysis by omitting one study each time. Begg's test and Egger's linear regression were used to provide statistical proof for the publication bias existence.

## 2. Results

### 2.1. Study Selection, Characteristics, and Quality Assessment

**Figure 1:**
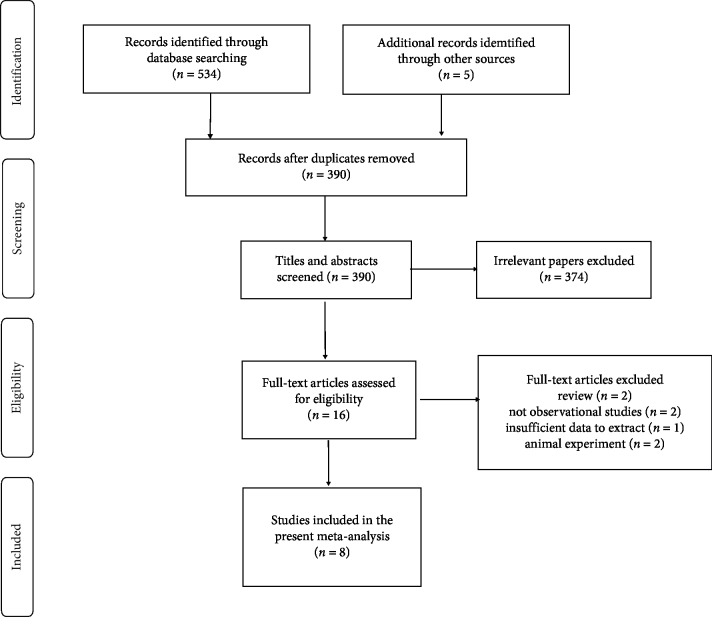
Study selection flow diagram.

The selection process of enrolled studies is shown in Figure 1. The articles included were published before April 2019. Study characteristics of the observational studies included and study outcomes are listed in Table 1; Table 2 lists the data for the association of urolithiasis and atherosclerosis from each inclusion study, and [Supplementary-material supplementary-material-1] lists the characteristics of the study samples which were included in the meta-analysis. Seven articles [20–23, 25–27] were published in the full form, and one [24] was in the abstract form. The eight observational studies [20–27] included 70,716 samples. Among them, three of them were performed in the United States. Five studies originated from Korean, UK, Italian, Japan, and Saudi Aljouf, respectively. The OR values and unadjusted 95% confidence interval can be calculated by STATA software (Table 1). A total of five studies have adjusted values [20–23, 26].

The detailed risk assessment information is shown in Tables [Supplementary-material supplementary-material-1]–[Supplementary-material supplementary-material-1].

According to the NOS (Newcastle–Ottawa scale) and AHRQ scale, one case-control study was considered to have poor quality and then the remaining studies were considered to have fair to good quality (Tables [Supplementary-material supplementary-material-1]–[Supplementary-material supplementary-material-1]).

### 2.2. Main Analysis between Urolithiasis and Atherosclerosis

**Figure 2:**
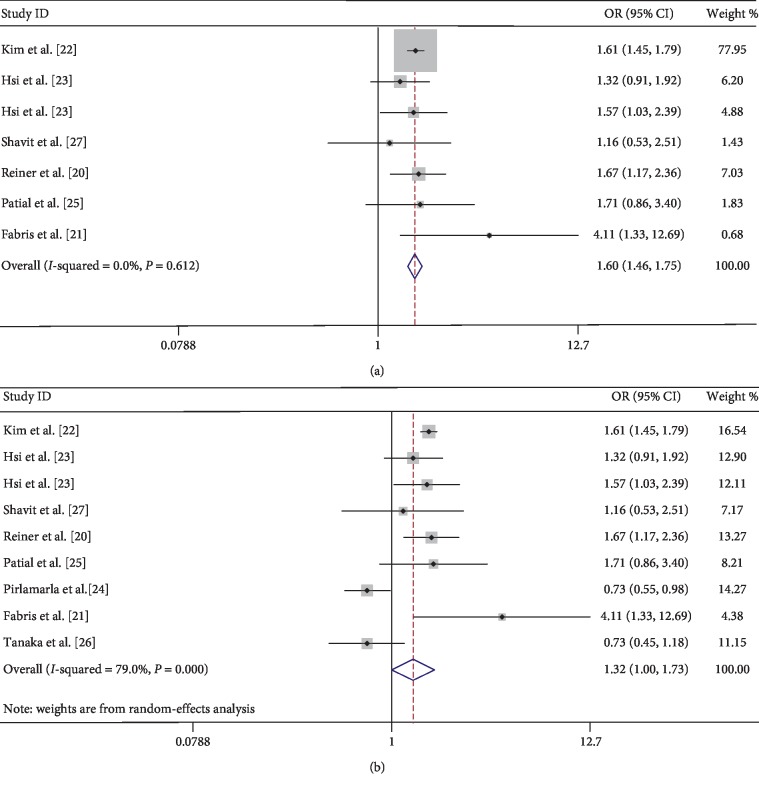
Forest of comparison: urolithiasis versus without urolithiasis and event: arteriosclerosis. OR value and CI without adjusting potential confounding factors. (a) Removal of heterogeneous sources: Pirlamarla et al. [24]. (b) Without removal of heterogeneous sources: Pirlamarla et al. [24]. OR: odds ratio; CI: confidence interval.

**Figure 3:**
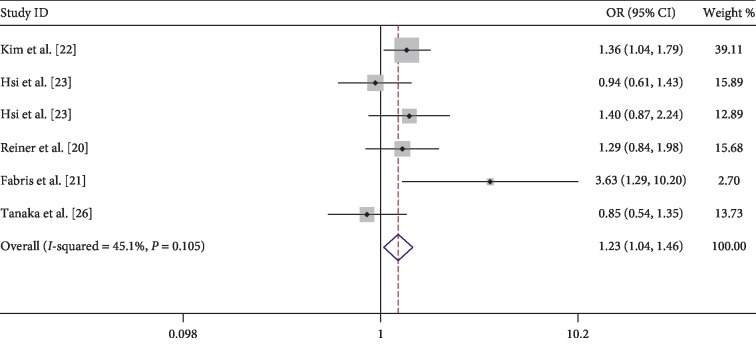
Forest of comparison: urolithiasis versus without urolithiasis and event: arteriosclerosis. OR value and CI adjusting potential confounding factors. OR: odds ratio; CI: confidence interval.

**Figure 4:**
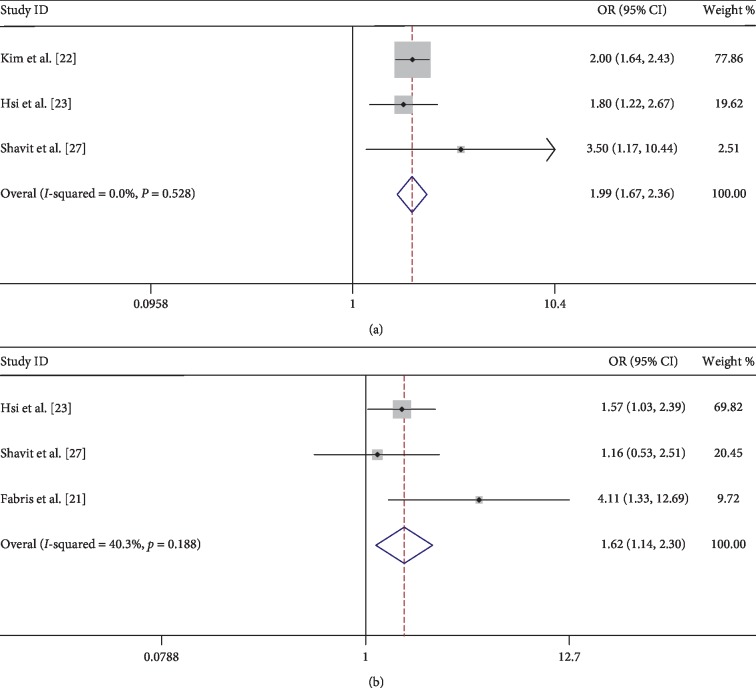
(a) Forest of comparison: renal calculi versus without renal calculi and event: moderate or severe arteriosclerosis. (b) Forest of comparison: recurrent renal calculi versus without renal calculi and event: arteriosclerosis. OR: odds ratio; CI: confidence interval.

The eight observational studies, with 70,716 samples, proved the correlation between urolithiasis and arteriosclerosis. According to the first merged data without adjusting potential confounders, the incidence of arteriosclerosis did increase in patients with urolithiasis, compared with those who did not have urolithiasis (OR = 1.32; 95% CI = 1.00–1.73; *z* = 1.96; *P* = 0.05), but the heterogeneity was significant (*I*^2^ = 79%, *P* < 0.001) (Figure 2(b)). The multivariate-adjusted correlation between urolithiasis and arteriosclerosis for the five studies is presented in Figure 3, according to the heterogeneity test (*I*^2^ = 45.1%, *P* = 0.105), and a fixed-effects model was used to evaluate the correlation between urolithiasis and arteriosclerosis. The results indicated that the incidence of arteriosclerosis would increase in patients with urolithiasis (OR = 1.23; 95% CI = 1.04–1.46; *z* = 2.38; *P* = 0.017) (Figure 3). Furthermore, the meta-analysis showed renal calculi increased the risk of moderate or severe arteriosclerosis (OR = 1.99; 95% CI = 1.67–2.36; *z* = 7.76; *P* < 0.001) with nonsignificant evidence of heterogeneity (*I*^2^ = 0.0%, *P* = 0.528) (Figure 4(a)) and also showed a significant increase in the proportion of patients with arteriosclerosis in patients with recurrent renal calculi in Figure 4(b) (OR = 1.62; 95% CI = 1.14–2.30; *z* = 2.69; *P* = 0.007) with modest evidence of heterogeneity (*I*^2^ = 40.3%, *P* = 0.188).

**Figure 5:**
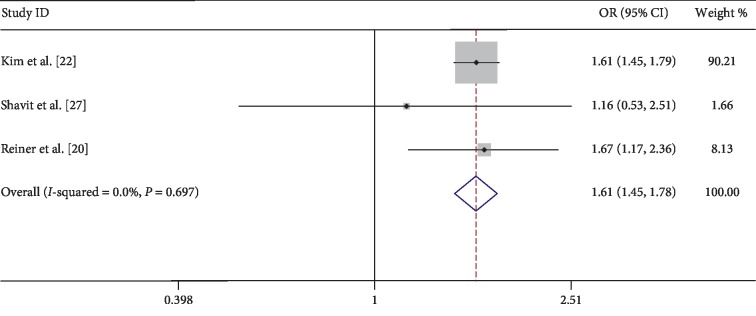
Forest of comparison: urolithiasis versus without urolithiasis and event: arteriosclerosis in people with normal uric acid. OR: odds ratio; CI: confidence interval.

Interestingly, we can still observe that urolithiasis is associated with a higher risk of atherosclerosis in people with normal uric acid (<6.0 mg/dl) in Figure 5 (OR = 1.61; 95% CI = 1.45–1.78; *z* = 9.28; *P* < 0.001) and with no evidence of heterogeneity (*I*^2^ = 0.0%, *P*=0.697) when we merge the data of 65,751 samples [20, 22, 27] (Figure 5).

### 2.3. Subgroup Analysis

When stratified by BMI, the pooled BMI > 28 kg/m^2^ studies showed a statistically significant association (OR = 1.53; 95% CI = 1.24–1.89; *z* = 4.02; *P* < 0.001; *n* = 4 studies) with nonsignificant heterogeneity (*I*^2^ = 0.00%, *P*=0.812), whereas the BMI < 28 kg/m^2^ studies showed no association (OR = 1.50; 95% CI = 0.83–2.71; *z* = 1.33; *P*=0.182; *n* = 3 studies) with significant heterogeneity (*I*^2^ = 79.7%, *P*=0.007) ([Supplementary-material supplementary-material-1]).

In the studies included, the definitions of the outcome of atherosclerosis were different, so a subgroup analysis of the outcome was carried out. The pooled coronary artery calcification studies and carotid atherosclerosis studies showed a statistically significant association (OR = 1.59; 95% CI = 1.44–1.75; *n* = 3 studies, and OR = 1.68; 95% CI = 1.23–2.29; *n* = 2 studies, respectively) with low heterogeneity for both subgroups (*I*^2^ = 0.00% and *I*^2^ = 0.00%, respectively) ([Supplementary-material supplementary-material-1]), whereas the abdominal aortic calcification studies and peripheral artery atherosclerosis studies showed no association (OR = 0.79; 95% CI = 0.56–1.13; *n* = 2 studies, and OR = 1.71; 95% CI = 0.37–7.93; *n* = 2 studies, respectively) with evidence of heterogeneity (*I*^2^ = 16.5% and *I*^2^ = 84.2%, respectively) ([Supplementary-material supplementary-material-1]).

### 2.4. Sensitivity Analysis and Publication Bias

For sensitivity analysis, one study was omitted at a time and the pooled OR values of the remaining studies were calculated. In these analyses, except for Pirlamarla et al.'s [24] research, the OR values were similar and do not fluctuate significantly, ranging from a low value of 1.33 (95% CI = 1.01–1.75) to a high value of 1.43 (95% CI = 1.07–1.92), respectively ([Supplementary-material supplementary-material-1]). After removing the heterogeneous sources of the research [24], we found that the heterogeneity of all analyses containing heterogeneous sources was significantly reduced. Heterogeneity decreased from *I*^2^ = 79% to *I*^2^ = 0.00% (Figures 2(b) versus 2(a)) and from *I*^2^ = 77.4% to *I*^2^ = 38.7% (Figures [Supplementary-material supplementary-material-1] versus [Supplementary-material supplementary-material-1]).

Sensitivity analysis revealed that Pirlamarla et al.'s [24] study is the source of heterogeneity in the meta-analysis for urolithiasis and arteriosclerosis. When this outlier study is removed, heterogeneity declined significantly in the remaining studies (*I*^2^ = 0.00%, *P*=0.612). The reasons why this study has become a heterogeneous source may be the following: It was a low-quality case-control study. Among the eight studies included, the lowest score obtained by the NOS was only 3 points. The study was a summary of the conference without a full text. So we did not know the specific settings of the experiment, the selection criteria of the control group and the experimental group, the follow-up time, the rate of lost visits, and so on. The study also did not provide potential confounders such as gender, age, BMI, energy intake, blood pressure tables, and blood sugar.

We conducted Begg's test and Egger's linear regression. The *P* value of Begg's test was 1.0, and the *P* value of Egger's linear regression was 0.528 (Table 3).

## 3. Discussion

The association between urolithiasis and metabolic syndromes [28, 29] (including hypercholesterolemia, insulin resistance [8], hyperglycemia, hypertension, obesity [10], and hyperuricemia) has been indicated in several studies. In this meta-analysis included in the study, Tanaka et al. study [26] mentioned that the evidence for an increased risk of arteriosclerosis in serum uric acid >7.0 mg/dl with urolithiasis is statistically significant (OR = 0.52; 95% CI = 0.27–0.99; *P*=0.047). As is known to all, high blood uric acid will increase kidney stone formation and the risk of cardiovascular disease. This mechanism may be one of the links between urolithiasis disease and atherosclerosis. But when we merged the data from 65,751 samples which provided normal uric acid values (serum uric acid <6.0 mg/dl) in the 3 articles [20, 22, 27], the results still showed that urolithiasis would increase the risk of atherosclerosis (OR = 1.61; 95% CI = 1.45–1.78; *z* = 9.28; *P* < 0.001).

In the present meta-analysis, whether the potential confounding factors were adjusted or not, urolithiasis would increase the risk of atherosclerosis. It would especially increase the incidence of coronary atherosclerosis and carotid atherosclerosis which is associated with the outcome of CV disease [30–34].

As is mentioned earlier, metabolic disorders, including obesity, may be the potential risk between urolithiasis and arteriosclerosis. In subgroups with BMI over 28 kg/m^2^, the incidence of atherosclerosis has a statistically significant increase in patients with urolithiasis. However, in our analysis, two articles [20, 22] with normal blood pressure and one article [20] with normal blood lipid profile showed an increased risk of atherosclerosis in patients with urolithiasis whether adjusting for confounding factors or not. These results suggested that urolithiasis may be an independent risk factor for atherosclerosis.

Urinary stone formation is a complex process, which is associated with uric acid, calcium, and phosphorus metabolism, oxalic acid salt, and so on. Studies have reported that 50% people with a history of kidney stones will have kidney stones recurred [35]. Glover et al. pointed out that the continuous development of kidney stones may lead to subsequent cardiovascular diseases [36]. Our analysis also found that recurrent kidney stones would increase the risk of atherosclerosis. This may be associated with repeated occurrences of calcium and pyrophosphate concentration [37]. Pyrophosphate is a phosphorus-oxygen anion, an inhibitor of calcification and crystallization, present in the heart and kidney, respectively. Inadequate or low concentrations of pyrophosphate can lead to arterial calcification and abnormal renal crystallization. Yasui et al. reported higher aortic calcification scores in patients with urolithiasis compared with a control group [38]. In our analysis, we also found that urinary calculi, especially renal calculi, can increase the severity of atherosclerosis. According to some studies, the severity of atherosclerosis is known as a powerful predictor of coronary and cerebrovascular diseases and death [27, 39].

The present meta-analysis has several advantages: First, it is the first meta-analysis of the relationship between urolithiasis and risk of atherosclerosis. Second, this meta-analysis contains 70,716 samples, so the conclusion based on this meta-analysis is valid. Third, with the combined data of adjusted potential confounding factors, it can minimize the confounding factors. In addition, the heterogeneity of meta-analysis in this paper is small. And there is no significant publication bias, and the impact on merged effect value can be neglected. Thus, the conclusion of this meta-analysis is reliable.

However, the present meta-analysis has several limitations: First, in the included observational studies, the quality assessment of the scale shows that not all observational studies are of high quality. One case-control study was considered to have poor quality, and then the remaining studies were considered to have fair to good quality. Second, the included observational studies have inherent limitations; thus, we cannot draw a conclusion about the relationship casually. We also cannot exclude residual confounding factors by inaccurately measured atherosclerosis factors or unmeasured confounding factors. Although this study analyzed the data adjusted for multiple potential confounding factors, the possibility of residual confounding due to increased risk characteristics of atherosclerosis and urolithiasis cannot be ruled out. Finally, in this meta-analysis, there are fewer articles providing normal blood pressure and lipid profile. So it is impossible to further explore whether urolithiasis is a risk factor for atherosclerosis independent of blood pressure and lipid. More prospective experiments are needed to prove the relationship and the possible mechanism between other urolithiasis and atherosclerosis.

In conclusion, the present meta-analysis of observational studies with 70,716 samples found that urolithiasis disease increased the risk of atherosclerosis, especially in coronary artery and carotid atherosclerosis. Furthermore, the significant effect was seen on normal uric acid. We can draw a conclusion that urolithiasis maybe is a potential risk factor of coronary atherosclerosis and carotid atherosclerosis, which is independent of uric acid. The underlying mechanism still needs further exploration.

## Figures and Tables

**Table 1:** Studies on urolithiasis and arteriosclerosis risk included in the meta-analysis.

Basic characteristics of included studies									
Study	Study design	Sex	Country	Sample size	Average age	Follow-up time	Outcome	Outcome measurement	Results
Kim et al. [22]	Cross-sectional study	Both (men and women)	Korean	62091	41.5	—	Coronary artery calcification (CAC)	CAC was assessed with a LightSpeed VCT XTe 64-slice multidetector CT scanner	The prevalence of detectable CAC was higher in participants with nephrolithiasis than those without nephrolithiasis (19.1% versus 12.8%).

Hsi et al. [23]	Cohort study	Both	United States	3276	69.5	2000–2012	Coronary artery calcification (CAC)	Multidetector row computerized tomography using a standardized protocol was performed on the participants	The study shows an independent association between a history of recurrent kidney stone formation and coronary artery calcium, specifically in participants with medium or high CAC scores.

Shavit et al. [27]	Case-control study	Both	United Kingdom	111	47	2011–2014	Abdominal aortic calcification	Obtained the CT images for aortic calcification using a standard radiology picture archiving and communication system workstation	The AAC severity score (presented as the median (25th percentile, 75th percentile)) was significantly higher in KSFs compared with the control group (0 (0, 43) versus 0 (0, 10), *P* < 0.001).

Reiner et al. [20]	Cohort study	Both	United States	3549	White: 45.6; African American: 44.5	Included from 1985 to 1986. Follow-up for 20 years	Carotid atherosclerosis	Carotid IMT was determined by B-mode ultrasound (GE LOGIQ 700) examination using standard procedures after 20 years of follow-up	The association of kidney stones with carotid atherosclerosis was significant (OR 1.6, 95% CI 1.1–2.3, *P*=0.01), even after adjusting for major atherosclerotic risk factors.

Patil et al. [25]	Present study	Both	Saudi Aljouf	240	Stone group: 40.6; control group: 41.1	—	Carotid artery calcification	Any radiopaque nodular mass adjacent to the cervical vertebrae inside or below the C3-C4 intervertebral disc level, or the retromandibular area, generally at an angle of 45° from the angle of the mandible independent of the hyoid bone was considered a CAC	No significant relationship was found between the presence of CAC in the patients with renal stones and the control group. However, there was a trend for higher prevalence of CAC in renal stone patients.

Pirlamarla et al. [24]	Case-control study	Both	United States	925	—	2004–2013	Abdominal aortic calcification (AAC)	VC was measured as abdominal aortic calcification (AAC) between L1 and L4 vertebrae on noncontrast CT images	AAC was present in 46% of KSFs and 54% of controls (*P*=0.02). Both AAC prevalence and AAC severity are greater in controls than KSFs.

Tanaka et al. [26]	Cohort study	Both	Japan	440	Stone group: 63; control group: 62	2010–2014	Aortic calcification	ACI was quantitatively measured using abdominal CT images above the common iliac artery bifurcation by scanning 10 times at 10-mm intervals	ACI was not significantly high in the stone group compared with the nonstone group.

Fabris et al. [21]	Cohort study	Both	Italian	84	—	—	Increased arterial stiffness	PWV measurements were obtained with PulsePen, a noninvasive portable device. The PWV was calculated as distance between the measurement sites divided by a transit time delay between radial and carotid pulse waves and expressed as meter per second (m/s)	The prevalence of AAS was higher among stone formers compared with nonstone formers (36 versus 12%, *P*=0.01), and the difference remained significant even after adjustment for potential confounders.

NA, not available; BMI, body mass index; ACI, aortic calcification index; CT, computed tomography; PWA, pulse-wave velocity; KSF, kidney stone former.

**Table 2:** Estimates for the association of urolithiasis and arteriosclerosis risk reported in the included studies.

Study	With urolithiasis		Without urolithiasis		Events for analysis	OR (95% CI)		Adjusted confounders
	Events	Total	Events	Total		Unadjusted	Multivariable	
Kim et al. [22]	451	2363	7645	59728	Coronary artery calcification (CAC)	1.61 (1.45–1.79)	1.36 (1.04–1.79)	Age, sex, center, year of screening examination, physical activity, alcohol intake, smoking status, education level, body mass index, family history of cardiovascular disease, total energy intake, glucose concentration, systolic blood pressure, triglyceride, high-density lipoprotein cholesterol, uric acid concentrations, and estimated glomerular filtration rate

Hsi et al. [23]	NA	NA	NA	NA	Coronary artery calcification (CAC)	1.32 (0.91–1.92)	0.94 (0.61–1.43)	Age, gender, race/ethnicity, diabetes status, daily energy intake, body mass index, animal protein consumption, calcium intake, and sodium intake
	NA	NA	NA	NA	Coronary artery calcification (CAC)	1.57 (1.03–2.39)	1.40 (0.87–2.24)	

Shavit et al. [27]	22	57	19	54	Abdominal aortic calcification (AAC)	1.16 (0.53–2.51)	—	Age, sex, high BP, diabetes, smoking status, and eGFR

Reiner et al. [20]	NA	NA	NA	NA	Presence of carotid stenosis and/or upper quartile of bulb/internal carotid IMT	1.67 (1.17–2.36)	1.29 (0.84–1.98)	Age, gender, race, clinic status, smoking, treated hypertension, systolic BP, BMI, LDL-cholesterol, HDL-cholesterol, eGFR, uric acid, and HOMA index

Patil et al. [25]	25	120	16	120	Carotid artery calcification	1.71 (0.86–3.40)	—	—

Pirlamarla et al. [24]	309	672	136	253	Abdominal aortic calcification (AAC)	0.73 (0.55–0.98)	—	—

Tanaka et al. [26]	NA	NA	NA	NA	Aortic calcification (iliac artery bifurcation)	—	0.85 (0.54–1.35)	Age, sex, BMI, presence of comorbidities, urine protein, CKD stage, and serum uric acid

Fabris et al. [21]	15	42	5	42	Abnormal arterial stiffness (AAS) (CR-PWV, CF-PWV above 90% of the sample distribution)	4.11 (1.33–12.69)	3.63 (1.29–10.20)	Age, sex, and body mass index (BMI); models with measures of arterial stiffness as dependent variables were further adjusted for MAP, PP, and heart rate (HR)

NA, not available; OR, odds ratio; CI, confidence interval; CR-PWV, carotid-radial pulse-wave velocity; CF-PWV, carotid-femoral pulse-wave velocity.

**Table 3:** Publication bias of Begg's test and Egger's linear regression.

*Begg's test*		
Adj. Kendall's score (P-Q) = 0		
Std. dev. of score = 9.59		
Number of studies = 9		
*z* = 0.00		
Pr > *z* = 1.000		
*z* = −0.10	(Continuity corrected)	
Pr > *z* = 1.000	(Continuity corrected)	

*Egger's test*		
Std. coeff. std. err.	*t P* > *t* (95% CI)	
Slope 0.4527417 0.1526158	2.97 0.021 0.0918628	0.8136207
Bias −0.7614662 1.147271	−0.66 0.528–3.474332	1.951399

## Supplementary Materials

Supplementary Materials Figure S1: outcome subgroup analysis. (a) Forest of comparison: urolithiasis versus without urolithiasis and event: arteriosclerosis with removal of heterogeneous sources: Pirlamaral et al. [24]. (b) Forest of comparison: urolithiasis versus without urolithiasis and event: arteriosclerosis without removing heterogeneous sources: Pirlamaral et al. [24]. OR: odds ratio; CI: confidence interval. Figure S2: sensitivity analyses of association between urolithiasis and risk of arteriosclerosis. Figure S3: BMI subgroup analysis. Forest of comparison: urolithiasis versus without urolithiasis and event: arteriosclerosis. OR: odds ratio; CI: confidence interval. Table S1: Newcastle–Ottawa scale for assessment of quality cohort studies. Table S2: Newcastle–Ottawa scale for assessment of quality case-control studies. Table S3: cross-sectional/prevalence study quality. Table S4: basic characteristics of the study sample included in this meta-analysis on urolithiasis and arteriosclerosis risk.
