# Supplementary material for: Urolithiasis, Independent of Uric Acid, Increased Risk of Coronary Artery and Carotid Atherosclerosis: A Meta-Analysis of Observational Studies
Source: Biomed Res Int. 2020 Feb 20;2020:1026240. doi: 10.1155/2020/1026240 (PMC7053446; doi:10.1155/2020/1026240)
Supplement: Supplementary Materials — Figure S1: outcome subgroup analysis. (a) Forest of comparison: urolithiasis versus without urolithiasis and event: arteriosclerosis with removal of heterogeneous sources: Pirlamaral et al. [24]. (b) Forest of comparison: urolithiasis versus without urolithiasis and event: arteriosclerosis without removing heterogeneous sources: Pirlamaral et al. [24]. OR: odds ratio; CI: confidence interval. Figure S2: sensitivity analyses of association between urolithiasis and risk of arteriosclerosis. Figure S3: BMI subgroup analysis. Forest of comparison: urolithiasis versus without urolithiasis and event: arteriosclerosis. OR: odds ratio; CI: confidence interval. Table S1: Newcastle–Ottawa scale for assessment of quality cohort studies. Table S2: Newcastle–Ottawa scale for assessment of quality case-control studies. Table S3: cross-sectional/prevalence study quality. Table S4: basic characteristics of the study sample included in this meta-analysis on urolithiasis and arteriosclerosis risk. [file 1026240.f1.pdf]

Figure S1

Figure S1A

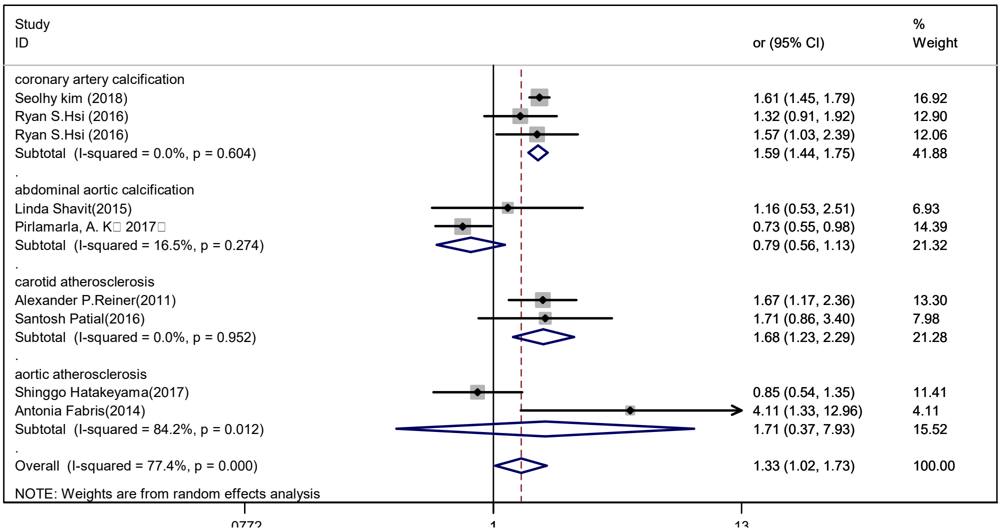

Figure S1B

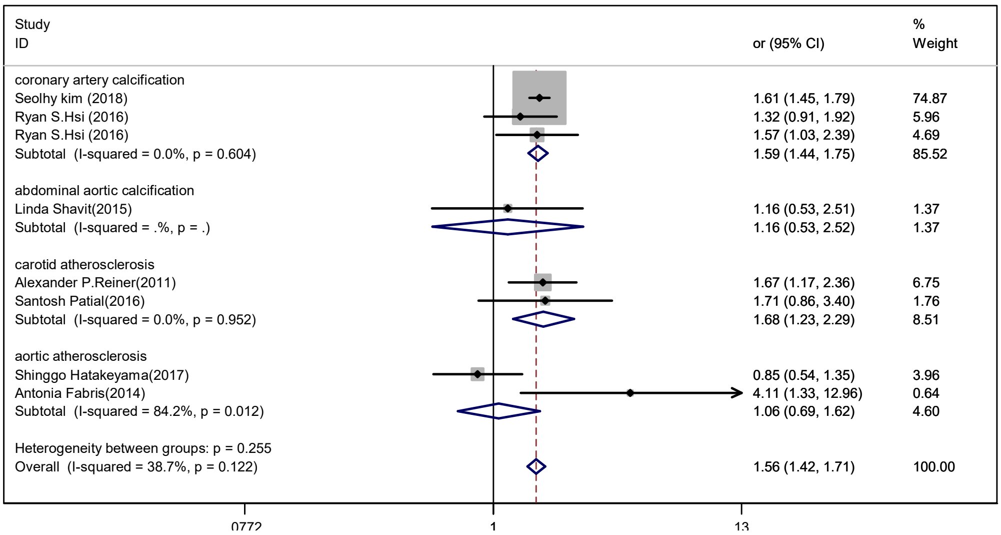

Figure S2

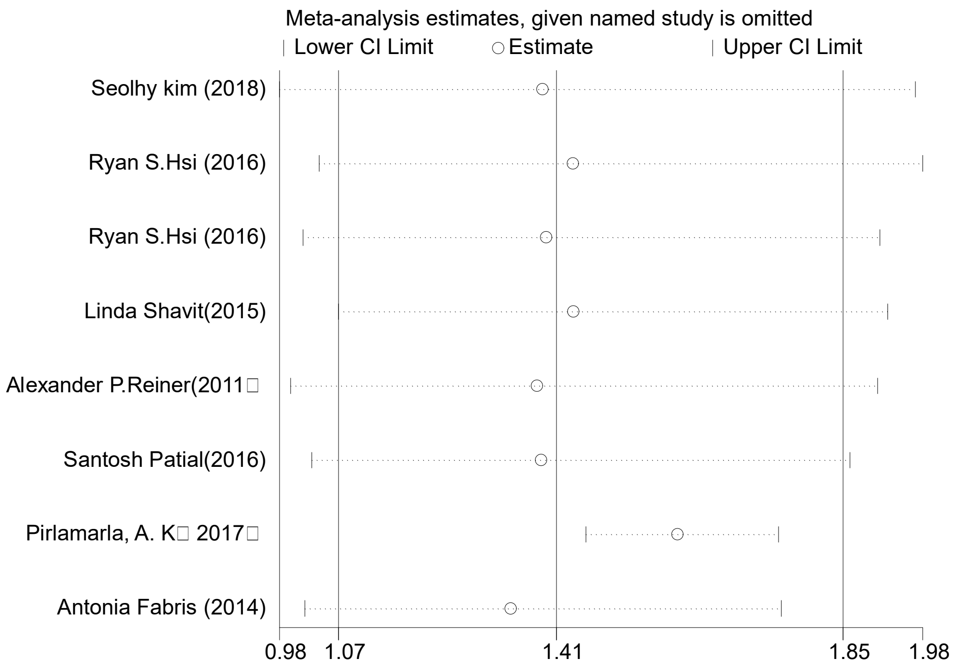

Figure S3

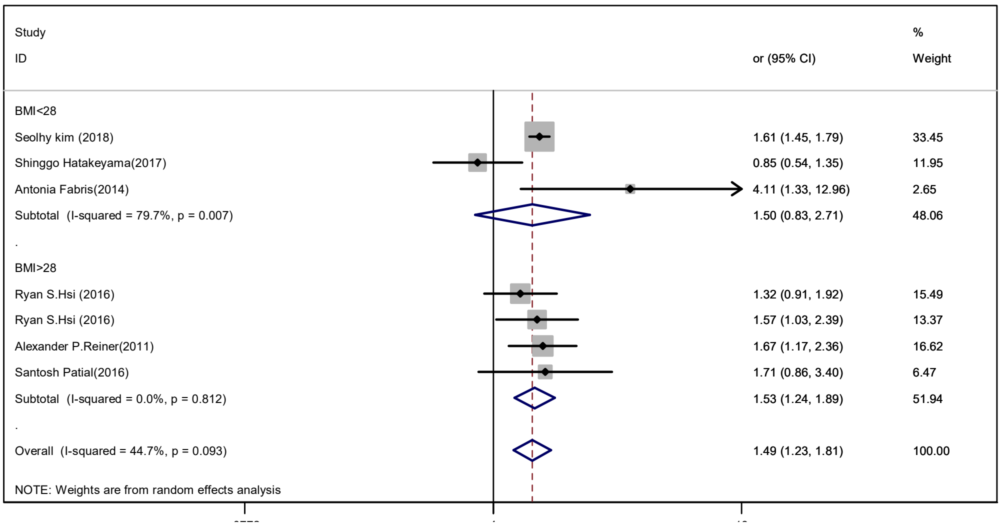

Newcastle-Ottawa Scalefor Assessment of Quality Cohort studies

			Ryan S.Hsi (2016)	Alexander P.Reiner（2011）	Shinggo Hatakeyama(2017)	Antonia Fabris(2014)
Quality Assessment Criteria	Selection	Representativeness of the exposed cohort?	1	1	1	1
		Representativeness of the nonexposed cohort?	1	0	0	0
		Ascertainment of exposure?	1	0	1	0
		Affirming the starting trend of research without observing the outcome indicators	1	0	0	0
	Comparability	Study controls for age and sex?	1	1	1	1
		Study controls other additional risk factors?	1	1	1	1
	Outcome	Assessment of outcome?	1	1	1	1
		Was follow-up long enough for outcomes to occur?	0	1	1	0
		Adequacy of follow-up of cohorts?	1	1	1	0
	Overall quality score		8	6	7	4

Table.S1 Newcastle-Ottawa Scale for Assessment of Quality Cohort studies.

[a] Each 1 point indicates individual criterion within the subsection was fulfilled.

Newcastle-Ottawa Scalefor Assessment of Quality Case-Control studies case-control studies

		Linda Shavit(2015)	Pirlamarla, A. K.（2017）
Selection	Is case selection appropriate?	1	1
	Representativeness of cases	1	0
	Control options	0	0
	Determination of control	0	0
Comparability	Study controls for age and sex?	1	0
	Study controls other additional risk factors?	1	0
Exposure factor measurement	Determination exposure factor	1	1
	The same method was used to determine the exposure factors of case and control group.	1	1
	Non response rate	0	0
Overall quality score		6	3

Table .S2 Newcastle-Ottawa Scale for Assessment of Quality Case-Control studies.
[a] Each 1 point indicates individual criterion within the subsection was fulfilled.

Cross-Section/Prevalence Study Quality

	Seolhye Kim (2018)	Santosh Patil(2016)
1)Define the source of information(survey,record review)	1	1
2)List inclusion and exclusion criteria for exposed and unexposed subjects(cases and controls)or refer to previous publications	1	1
3)Indicated time period used for identifing patients	1	0
4) Indicate whether or not subjects were consecutive if not population-based	1	0
5) Indicate if evaluators of subjective components of study were masked to other aspects of the status of the participants	0	1
6) Describe any assessments undertaken for quality assurance purposes (e.g., test/retest of primary outcome measurements)	1	1
7) Explain any patient exclusions from analysis	1	1
8) Describe how confounding was assessed and/or controlled.	1	1
9) If applicable, explain how missing data were handled in the analysis	1	0
10) Summarize patient response rates and completeness of data collection	1	1
11) Clarify what follow-up, if any, was expected and the percentage of patients for which incomplete data or follow-up was obtained	0	0
Overall quality score	9	7

Table .S3 AHRQ for Assessment of Quality Cross-Section/Prevalence Study

An item would be scored ‘0’ if it was answered ‘NO’ or ‘UNCLEAR’; if it was answered ‘YES’, then the item scored ‘1’.

		woman	man	m/W	n	age	BMI	SP	DP	eGFR(ml/min/1.73 m2)	Glucose(mg/dl)	serum creatinine (mg/dl)	Uric acid(mg/dl)	LDL(mg/dl)	HDL(mg/dl)	Triglycerides(mg/dl)
Seolhy kim (2018)	kidney stone	345	2018	5.84	2363	43.5±7.6	24.9±3.1	114.4±12.5	75±10.0	94.3±13	99.6±17.5	NA	6.0+1.4	131.3±32.9	53.1±13.3	120(86-172)
	non kidney stone	13380	46348	3.46	59728	41.4±7.6	24.2±3.2	112.1±12.6	73±10.0	97.3±13	97.7±16.2	NA	5.7+1.4	127.5±31.9	54.6±14	112(78-163)
	overall	13725	48366	3.52	62091	41.5±7.6	24.3±3.2	112.2±12.6	73.1±10	97.2±13	97.7±16.2	NA	5.7±1.4	127.6±32	54.5±14	112(78-163)
Ryan S.Hsi (2016)	single kidney stone	58	90	1.55	148	71.1±8.67	28±4.7	NA	NA	NA	NA	0.93±0.28	NA	NA	NA	NA
	recurrent kidney stone	38	91	2.39	129	68.9±8.84	29.7±5.3	NA	NA	NA	NA	0.96±0.29	NA	NA	NA	NA
	no kidney stone	1632	1376	0.84	2999	69.4±9.3	28.6±5.6	NA	NA	NA	NA	0.92±0.44	NA	NA	NA	NA
	overall	1728	1557	0.87	3276			NA	NA	NA	NA	NA	NA	NA	NA	NA
Linda Shavit(2015)	kidney stone	25	32	1.28	57	47±14	NA	NA	NA	91±22	88±18	1±0.4	5.5±1.3	NA	NA	NA
	non kidney stone	23	31	1.37	54		NA	NA	NA	91±15	93±18	0.9±0.1	NA	NA	NA	NA
	overall	48	63	1.31	111											
Alexander P.Reiner（2011）	overall white	1009	889	0.88	1898	45.6	27.9±6.5	NA	70±11	90.9±19.3	96±22	NA	5.67±1.43	110±31	54±17	119±88
	overall African American	1005	646	0.64	1651	44.5	31.3±7.6	121±16	77±12	104.7±24.7	100±31	NA	5.80±1.56	110±34	54±16	98±68
	overall all	2014	1535	0.76	3549											
Santosh Patial(2016)	kidney stone	44	76	1.72	120	40.6±7.8	28.6±4.8	NA	NA	NA	NA	NA	NA	NA	NA	NA
	no kidney stone	52	68	1.3	120	41.1±6.7	27.1±4.6	NA	NA	NA	NA	NA	NA	NA	NA	NA
Shinggo Hatakeyama(2017)	kidney stone	123	169	1.37	292	63	25±4	NA	NA	76	NA	NA	NA	NA	NA	NA
	no kidney stone	59	89	1.51	148	62	24.3±4	NA	NA	72	NA	NA	NA	NA	NA	NA
Antonia Fabris(2014)	kidney stone	23	19	0.83	42	42.6	24	NA	NA	NA	NA	0.82	NA	NA	NA	NA
	no kidney stone	25	17	0.68	42	40	24	NA	NA	NA	NA	NA	NA	NA	NA	NA

Table.S4 The basic characteristics of the studies sample included in this Meta- analysis on Urolithiasis and Arteriosclerosis Risk

a NA, not available;BMI,body mass index;SP,systolic blood pressures;DP,diastolic blood pressures;eGFR, glomerular filtration rate;LDL,low density lipoprotein cholesterol;HDL,high density lipoprotein cholesterol.
